# *Welwitschia*: Phylogeography of a living fossil, diversified within a desert refuge

**DOI:** 10.1038/s41598-021-81150-6

**Published:** 2021-01-27

**Authors:** Norbert Jürgens, Imke Oncken, Jens Oldeland, Felicitas Gunter, Barbara Rudolph

**Affiliations:** Institute of Plant Sciences and Microbiology, Team Biodiversity, Evolution and Ecology of Plants (BEE), University of Hamburg, Ohnhorststrasse 18, 22609 Hamburg, Germany

**Keywords:** Population genetics, Ecology

## Abstract

*Welwitschia mirabilis* is one of the most extraordinary plant species on earth. With a fossil record of 112 My and phylogenetically isolated within the order Gnetales, the monotypic genus *Welwitschia* has survived only in the northern Namib Desert in Angola and Namibia. Despite its iconic role, the biogeography, ecological niche, and evolutionary history of the species remain poorly understood. Here we present the first comprehensive map of the strongly disjunct species range, and we explore the genetic relationships among all range fragments based on six SSR markers. We also assess the variation of the environmental niche and habitat preference. Our results confirm genetic divergence, which is consistent with the hypothetical existence of two subspecies within *Welwitschia*. We identify an efficient geographical barrier separating two gene pools at 18.7°S in northern Namibia. We also identify further diversification within each of the two subspecies, with several different gene pools in ten isolated range fragments. Given the presence of well-isolated populations with unique gene pools and the association with different bioclimatic variables, rock types, and habitats within arid river catchments, we can hypothesize that the present intraspecific diversity may have evolved at least in part within the present refuge of the northern Namib Desert.

## Introduction

The phylogeny, morphology, and ecology of *Welwitschia mirabilis* make it one of the most extraordinary plant species on Earth. With a fossil record of 112 My and phylogenetically isolated within the order Gnetales, the genus *Welwitschia* is appropriately called a “living fossil.” Shortly after the discovery of *Welwitschia* by the Austrian botanist Friedrich Welwitsch, Hooker^1^ recognized similarities to conifers and proposed placing the new genus next to *Gnetum* L and *Ephedra* L. The most recent reviews based on matK and rbcL gene sequences validate Hooker’s proposal by revealing *Gnetum* as the closest relative of *Welwitschia* and *Ephedra* as sister to both^2^. The Gnetales are an extremely heterogeneous taxonomic group with an isolated phylogenetic position among seed plants. Relatives are known only from fossil records, mainly of Aptian age and scattered around the world (China, Russia, Europe, North and South America)^3^. Numerous Welwitschiaceae with close similarities to the present-day *Welwitschia* morphology occurred in northeast Brazil during the Aptian–Albian^3^ and were still connected to Africa^4^. These Welwitschiaceae coexisted with a diverse flora with no explicit xerophytic character and even including aquatic species^3^. The fossil record does not indicate whether major adaptations evolved while Welwitschia was limited in distribution to the Namib region, probably during the cooling and aridification of the Tertiary and Quaternary^3,5,6^. The phylogenetic age and isolation, the diversity of extinct relatives, and the huge biogeographical space allow us to regard the extant *Welwitschia* as a living fossil.

Leuenberger^7^ recognized two subspecies of *W. mirabilis ssp. namibiana* from Namibia and the typical *ssp. mirabilis* from Angola. Leuenberger observed morphological differences in the male strobili (cone length, color and wax cover, surface sculpturing, bract morphology and connation, peduncle length) and allopatric distribution of greenhouse plants of the dioecious species cultivated under identical conditions. Jacobson et al.^8^ questioned the validity of the two subspecies but studied only populations belonging to the Namibian subspecies. Therefore, their results neither support nor reject Leuenberger’s proposal.

Jacobson and Lester^9^ used random amplified polymorphic DNA markers (RAPD) to explore smaller-scale genetic diversity among populations in the central Namib Desert in Namibia and in one population from near Khorixas. The evidently minimal gene flow between populations separated by as little as 18 km implies limited transport of the pollen and seeds. This view is supported by Wetschnig and Depisch^10^, who reported mainly insect pollination and almost no longer-distance transport by wind.

Exploration of the geographical range of *Welwitschia* started almost simultaneously in Angola and Namibia. In Angola, Friedrich Welwitsch found the first plants on 3 September 1859 at the type locality on the coastal plateau near Cabo Negro (15.67°S, 11.92°E), 15 km NE of Tombua^1^. On 5 May 1861, the artist Thomas Baines found *Welwitschia* in Namibia in a dry riverbed near Heigamkab (22.69°S, 14.89°E). In Kew in 1863, Joseph Dalton Hooker inspected material from both collectors and published *Welwitschia mirabilis,* based on the type specimen from Angola^1^.

Subsequently, many authors reported new locations. In 1967, Kers^11^ visited sites in Namibia and published all records of that time. The southernmost record was reported from 23.6°S near Homeb. For Angola, Kers based the map records on Taborda de Morais^12^, who mentioned a population 8 km NE of the Rio de Sao Nicolau (= Rio Bentiaba), which is at 14.26°S. Jacobson et al.^8^ reported a N limit south of the Bentiaba River, while Henschel et al.^13^ reported 14.9°S, which is close to Moçâmedes (Namibé) at the latitude of Moscas Bay/Baba. Kers^11^ interpreted the biogeography as disjunct: “Most of the localities are so clearly separated from each other geographically that it is not even possible to imagine a gene exchange between them.” Giess^14^ published a few additional locations.

In summary, *Welwitschia* is presently confined to the northern part of the Namib Desert, which had an arid to semiarid climate during the whole Quaternary and most of the Tertiary^15^. Jacobson and Lester^9^ proposed that *Welwitschia* may have established the present range more than 105 My ago.

*Welwitschia mirabilis* has a long life expectancy^16,17^ and a highly unusual morphology. The stem diameter often surpasses 150 cm, but the stem height rarely surpasses 30 cm.

Hooker^1^ and, more recently, Cooper-Driver^18^ have already described the root morphology. The stem shows a continuous transition into a broad taproot that rapidly gets thinner with depth within the first meter. Further away from the stem, one to four smaller roots of only 1–3 cm diameter typically spread horizontally but also exploit deeper soil layers. Kutschera et al.^19^ found roots with horizontal lengths of 15 m. After germination, young plants rapidly develop a taproot. Several authors^17,20^ have interpreted *Welwitschia* as a phreatophyte based on the preferred habitat adjacent to drainage lines and the pronounced taproot formation. However, Bornman^21^ reported that roots near the Brandberg do not extend deeper than 3 m.

A recent study^13^ details the root architecture and water uptake of plants near the Swakop River. No roots deeper than 140 cm were observed, and the majority exploited rainwater within a gypsum crust and deeper petrocalcitic horizons. Only a small percentage showed upward growth, indicating uptake of a smaller amount of topsoil water. The same study presents evidence based on stable isotope analysis for a preferential water origin from continental water sources (= rare summer thunderstorms). Atlantic sources (= fog or winter rains) and water from gypsum crusts were much less relevant. Similarly, a study at Gobabeb^22^ did not show an influence of fog on leaf growth rate or germination.

Based on current knowledge of the range and phylogenetic position, we seek to establish a new and complete biogeographical record. Additionally, we explore the genetic similarities among seventeen range-wide populations using the first developed SSR markers for *Welwitschia*. It is remarkable that beyond the foci of the aforementioned studies and around 300 other publications^22^, there has been no attempt to capture the environmental niche of *Welwitschia* within the whole range (but see^23^ for some partial range elements). Therefore, our objectives are to (1) investigate genetic differentiation among the sample records from all range fragments in Angola and Namibia to determine whether they support the existence of two subspecies; (2) examine environmental differences among the geographic fragments; and (3) discuss the results that most obviously characterize the ecological niche of *Welwitschia*.

## Results

**Figure 1:**
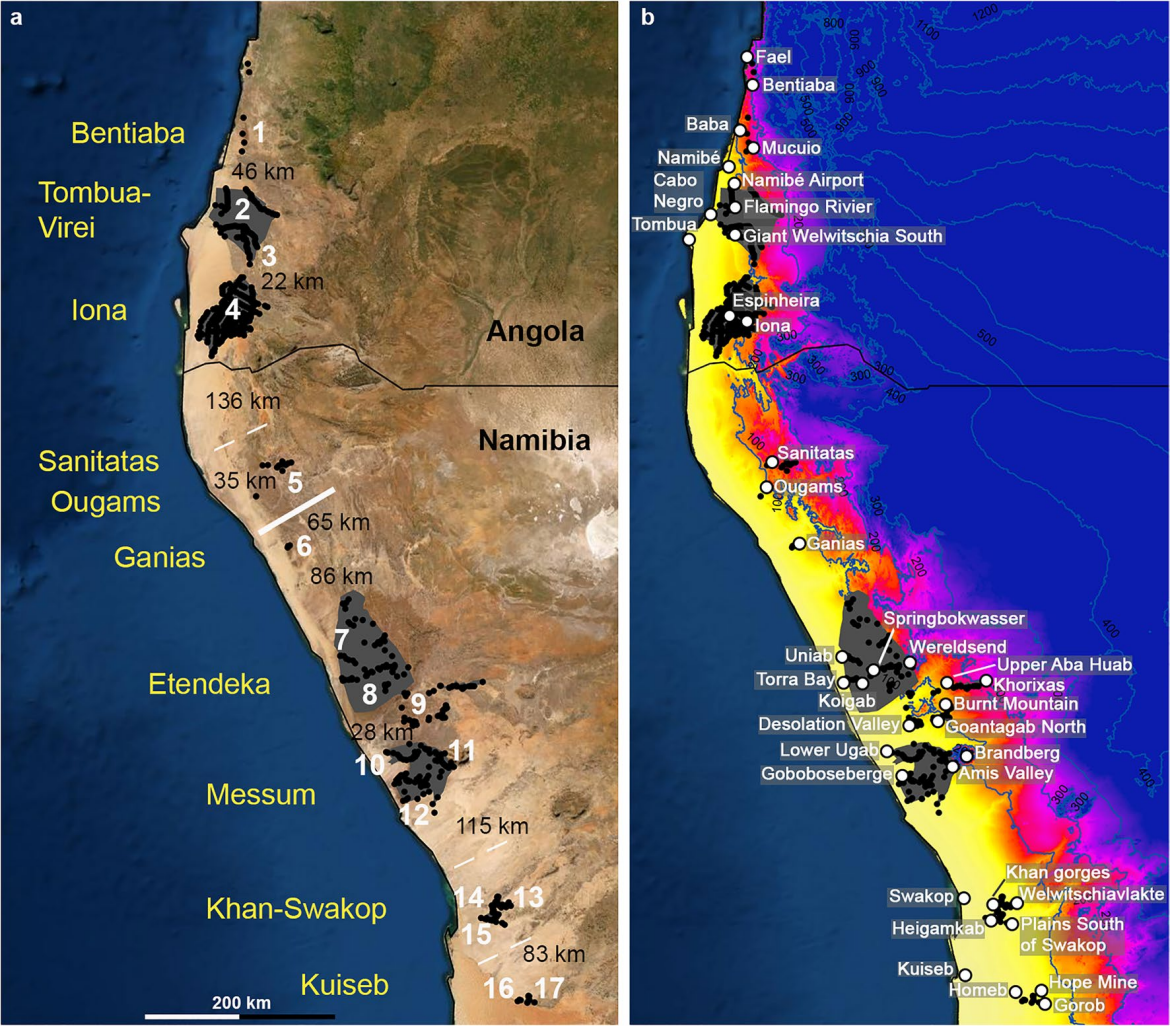
Disjunct distribution area of *Welwitschia* and its relation to annual rainfall. (**a**) Is a satellite map of southwestern Africa from 12.5° to 24°S latitude. This map was created using ArcGIS V.10.4.1. (www.esri.com). The black dots represent 991 *Welwitschia* locations confirmed by or newly recorded during this project. Large populations with a quasi-continuous presence are shown in gray. Isolated range fragments are named in yellow. White numbers and names designate the locations of populations sampled for SSR analysis. The isolation of range fragments is indicated by their distance in km. The white bar designates a major geographic gene-flow barrier; within *Welwitschia*, the broken lines designate geographic gene-flow barriers within subspecies. The black line marks the border between Angola and Namibia. (**b**) Shows *Welwitschia* locations and range fragments (as in **a**) against a color ramp, which illustrates the arid zone in yellow, orange, and red^24^. The blue lines are isohyets of mean annual rainfall in 100-mm increments. The horizontal black line marks the border between Angola and Namibia. The white dots refer to all locality names mentioned in the text and tables.

Our systematic mapping results allow for drawing a new map of the range of *Welwitschia* (Fig. 1). A total of 915 *Welwitschia* locations is derived from own observations, while 87 locations were compiled from herbaria and published literature. The resulting 991 confirmed locations are shown in Fig. 1. We also mapped landscapes with a continuous presence of *Welwitschia*. Based on the mapping, we could also group and name all the resulting range fragments (Table 1). The total extension of *Welwitschia mirabilis* measures 1096 km in an NNW–SSE direction (= 1.057 km latitudinal distance). The broadest section in a W–E direction connects populations located just west of Khorixas at -20.35° latitude to populations east of Torra Bay, 149 km further west. With the newly found locations, the area of distribution of the genus almost doubles in Angola. New northernmost, southernmost, and easternmost records have been made as well as new populations closer to the Atlantic coast than previously recorded. We found a new northernmost record in Angola, north of Fael at -14.12° latitude. Similarly, the southernmost record could be extended slightly to -23.63° latitude.

**Table 1:** *Welwitschia mirabilis* samples from Angola and Namibia used for analyses. This table is listing all sampled populations, which in several cases were composed of several geographically nearby subunits, with the exact locality information, the name of the range fragment and nearest neighbor distance between individuals of the closest subunit towards South.

Subspecies	Country	Population no	Population name	Samples no	Subunit of sample	Sample locality	Range fragment	Distance [km]	MAP [mm]	MAT [°C]	Coordinates
*W. m. ssp. mirabilis*	Angola	1	Moçâmedes-Bentiaba	14	1.1	Fael	Bentiaba	50	182	25.49	S14.12, E12.41
	Angola	1	Moçâmedes-Bentiaba		1.2	Mucuio	Bentiaba	46	162	24.95	S14.92, E12.37
	Angola	1	Moçâmedes-Bentiaba		1.3	Namibé Airport South	Tombua-Virei	< 1	87	24.48	S15.34, E12.20
	Angola	2	Tombua-Virei West	34	2	Flamingo Rivier	Tombua-Virei	< 1	72	24.37	S15.56, E12.14
	Angola	3	Tombua-Virei South	17	3	Giant Welwitschia South	Tombua-Virei	22	109	24.54	S15.90, E12.37
	Angola	4	Espinheira	25	4.1	Espinheira	Iona	< 1	111	23.05	S16.79, E12.36
	Angola	4	Iona Espinheira to Foz		4.2	Iona Espinheira to Foz	Iona	136	99	21.88	S16.96, E12.11
	Namibia	5	Kaoko	23	5.1	Sanitatas	Sanitatas	35	131	20.56	S18.22, E12.71
	Namibia	5	Kaoko		5.2	Ougams	Ougams	65	63	20.05	S18.51, E12.49
*W. m. ssp. namibiana*	Namibia	6	Ganias	7	6	Ganias	Ganias	86	66	19.75	S19.00, E12.84
	Namibia	7	Uniab	14	7	Uniab	Etendeka	< 1	42	20.29	S20.12, E13.36
	Namibia	8	Palmwag	16	8.1	Wereldsend	Etendeka	< 1	120	21.14	S20.23, E13.89
	Namibia	8	Palmwag		8.2	Springbokwasser	Etendeka	< 1	61	20.96	S20.33, E13.61
	Namibia	8	Palmwag		8.3	Koigab	Etendeka	< 1	53	20.55	S20.37, E13.52
	Namibia	8	Palmwag		8.4	Upper Aba Huab	Etendeka	28	120	22.57	S20.45, E14.46
	Namibia	9	Twyfelfontein	17	9,1	Burnt Mountain	Etendeka	< 5	113	22.16	S20.63, E14.42
	Namibia	9	Twyfelfontein		9.2	Goantagab North	Etendeka	< 20	100	22.35	S20.70, E14.39
	Namibia	9	Twyfelfontein		9.3	Desolation Valley	Etendeka	< 20	70	21.67	S20.82, E14.12
	Namibia	10	Ugab	12	10.1	Lower Ugab	Messum	< 20	28	20.57	S21.10, E13.82
	Namibia	10	Ugab		10.2	South Ugab plains	Messum	< 20	44	20.73	S21.18, E14.02
	Namibia	10	Ugab		10.3	Ugab hills	Messum	< 20	55	21.45	S21.17, E14.08
	Namibia	11	Brandberg	22	11	Amis Valley Brandberg apron	Messum	< 20	81	21.85	S21.45, E14.43
	Namibia	12	Goboboseberge	12	12	Goboboseberge	Messum	115	22	20.27	S21.37, E13.94
	Namibia	13	Welwitschiavlakte	42	13	Welwitschiavlakte	Khan-Swakop	< 5	46	20.49	S22.65, E14.99
	Namibia	14	Khan gorges	20	14	Khan gorges	Khan-Swakop	< 5	35	20.77	S22.66, E14.89
	Namibia	15	Plains S of Swakop	16	15	Plains S of Swakop	Khan-Swakop	83	31	20.12	S22.76, E14.94
	Namibia	16	Homeb	18	16	Homeb	Kuiseb	9 (towards E)	37	21.01	S23.61, E15.17
	Namibia	17	Kuiseb East	20	17.1	Hope Mine	Kuiseb	6 (towards SE)	47	20.79	S23.57, E15.26
	Namibia	17	Kuiseb East		17.2	Gorob	Kuiseb		47	21.19	S23.64, E15.32

The new northernmost record is nearest to the coast, 4.8 km inland from the beach at Fael. From N to S, the other large range fragments have the following minimum distances to the coast: 11.1 km (Tombua-Virei, record near Flamingo), 12.6 km (Iona), 13.0 km (Etendeka, record inland of Torra Bay), 8.5 km (Messum, record at Goboboseberge), 29.9 km (Khan–Swakop), 53.0 km (Kuiseb).

The *Welwitschia* population farthest from the Atlantic coast (149 km) is situated just W of Khorixas at -20.39 latitude. This is also the most humid site at just over 200 mm mean annual precipitation (MAP), compared to the majority of populations at less than 100 mm MAP.

The biogeographical range of *Welwitschia* is strongly fragmented into spatially isolated, disjunct partial ranges in a linear spatial arrangement from north to south along the hyperarid to arid Namib Desert. In this article, we distinguish 10 isolated (disjunct) “range fragments” using a 20-km distance threshold for assumed isolation, given the limited pollen transport of Diptera and Hymenoptera^10^ and the reported lack of gene flow^9^. The range fragments are presented in Fig. 1a and Table 1.

In both countries, we found aggregates of two large range fragments separated by gaps smaller than 30 km; the two in Angola (Tombua–Virei and Iona) are 309 km from the two in Namibia (Etendeka and Messum). In the space between them and in the extreme north and south of the whole range are additional small range fragments. The aggregate of the two Namibian range fragments, Etendeka and Messum, is larger in area, while the two Angolan range fragments, Tombua–Virei and Iona, contain the largest number of individual plants. The high number of individuals strongly correlates to the very high plant density in Tombua–Virei and Iona. In our standard plots of 1000 m^2^ we often counted more than 200 plants: for example, in plot 36,317 (-16.697, 12.432) in the northern part of Iona NP we found 212 individuals, while in plot 31,555 (-22.654, 14.987) in the Khan–Swakop range fragment we found only six individuals.

In a first approximation, populations of the genus *Welwitschia* are viable within an arid niche defined by a MAP of less than 200 mm; many fragments in parts receive less than 100 mm, and the more southern range fragments less than 50 mm (Fig. 1b, Table 1). At the arid extreme of the niche, the coastal Welwitschia records of the Messum fragment at Goboboseberge are just above 20 mm MAP.

With regards to the rock types (Table 2), granite, basalt, limestone, sandstone, and mica schist play an important role in different range fragments. A more constant factor is the preference of habitats on alluvial soils in or adjacent to small dry riverbeds and/or terraces bordering larger dry riverbeds. Rocky plains with shallow leptosols and rocky or stony slopes—especially at the lower end of a slope, often in sandy patches below granite boulders or other larger rock surfaces—are also found in some places. Calcretes are found in most of the range fragments, while gypcretes play an important role in the western parts of the Messum and Khan–Swakop range fragments.

**Table 2:** Environmental and habitat features of the sampling sites for 17 *Welwitschia* populations in Angola and Namibia.

Population sample	Locality of sample	Habitat	Substrate	Lithology	Geology	Population structure
1.1	Fael	Small dry riverbeds	Alluvial	Limestone and sandstone	Cretaceous	Scattered linear population
1.2	Mucuio	Small dry riverbeds	Alluvial	Limestone and sandstone	Cretaceous	Scattered linear population
1.3	Namibé Airport South	Small dry riverbeds	Alluvial	Limestone and sandstone	Pliocene to Quaternary	Continuous linear population
2	Flamingo River	Dry riverbed terraces	Alluvial	Limestone and sandstone	Pliocene to Quaternary	Continuous linear population
3	Giant Welwitschia South	Dry riverbeds and rocky plains	Alluvial to shallow leptosols	Granite	Archaean	Continuous planar population
4.1	Espinheira	Dry riverbeds and rocky plains	Shallow leptosols and alluvial	Limestone and sandstone	Paleogene	Continuous planar population
4.2	Iona Espinheira to Foz	Dry riverbeds and rocky plains	Shallow leptosols and alluvial	Limestone and sandstone	Paleogene	Continuous planar population
5.1	Sanitatas	Lower slopes and dry riverbanks	Alluvial and colluvium	Tillite, shale, sandstone	Dwyka	Irregularly scattered individuals
5.2	Ougams	Dry riverbeds	Alluvial	Marble, schist / tillit shale nearby	Karibib / Dwyka nearby	continuous Linear population
6	Ganias	Dry riverbeds and rocky plains	Shallow leptosols and alluvial	High-silica basalt	Etendeka	Continuous linear populations along riverbeds, scattered individuals in leptosol above basalt
7	Uniab	Dry riverbed terraces and lower rocky slopes	Shallow leptosols and alluvial	High-silica basalt	Etendeka	Continuous linear population
8.1	Wereldsend	Small dry riverbeds and plain	Shallow leptosols and alluvial	High-silica basalt	Etendeka	Continuous planar population
8.2	Springbokwasser	Small dry riverbeds and Plain	Shallow leptosols and alluvial	High-silica basalt	Etendeka	Scattered planar population
8.3	Koigab	dry riverbeds	Shallow leptosols and alluvial	High-silica basalt	Etendeka	Continuous linear population
8.4	Upper Aba Huab	Rocky slopes	Shallow leptosols and alluvial	High-silica basalt	Etendeka	Scattered planar population
9.1	Burnt Mountain	Rocky slopes	Shallow leptosols and rock fissures	Shale, mica schist	Karoo Damara	Continuous planar population
9.2	Goantagab North	Valley bottom, dry river terrace	Shallow leptosols and alluvial	Mica schist	Kuiseb	Continuous linear population
9.3	Desolation Valley	Small dry riverbeds	shallow leptosols and alluvial	Basalt, schist	Karoo Damara	continuous Linear population
10.1	Lower Ugab	Dry riverbed and sandy plain	Alluvial	Granite	Cambrian	Continuous linear population
10.2	South Ugab plains	Dry riverbed / valley bottom	Alluvial	Granite	Cambrian	Continuous linear population
10.3	Ugab hills	Dry riverbed / sheet wash valley bottom	Alluvial	Granite	Cambrian	Continuous linear population
11	Amis Valley Brandberg apron	Dry riverbed / sheet wash valley bottom	Alluvial	Mudstone, sandstone	Karoo	Continuous linear population
12	Goboboseberge	Dry riverbed/sheet wash valley bottom	Alluvial	Granite	Cambrian	Continuous linear population
13	Welwitschiavlakte	Small dry riverbeds	Alluvial	Sand, gravel, calcrete	Quaternary	Continuous planar population
14	Khan gorges	Dry riverbeds and lower rocky slopes	Alluvial	Gneiss	Mokolian	Continuous linear population
15	Plains S of Swakop	Dry riverbed/sheet wash plain	Alluvial	Quartzite	Damara	continuous Linear population
16	Homeb	Dry riverbed and adjacent slope	Alluvial	Mica schist	Damara	Continuous linear population
17.1	Hope Mine	Small dry riverbeds and plain	shallow leptosols and alluvial	Mica schist	Damara	Continuous linear population
17.2	Gorob	Small dry riverbeds and plain	Shallow leptosols and alluvial	Mica schist	Damara	Few scattered individuals

Concerning neighboring vegetation, *Welwitschia mirabilis* is most often associated with the following perennials (in decreasing order of importance): *Zygophyllum stapffii*, *Zygophyllum simplex*, *Arthraerua leubnitziae*, *Calicorema capitata*, *Petalidium variabile*, *Adenolobus pechuellii*, and *Commiphora wildii*^25^. In contrast, *Welwitschia mirabilis* is obviously outcompeted by *Acacia reficiens*, *Acacia mellifera*, *Acacia tortilis*, *Colophospermum mopane*, and sometimes *Salvadora persica*, as can be deduced from the immediate disappearance of *Welwitschia* at the margin of stands of these woody species—for example, when approaching Virei or Khorixas from the west. In contrast, among the more frequently disturbed vegetation of larger dry riverbeds, these species and *Welwitschia mirabilis* sometimes coexist, with old individuals growing next to each other, as in the case of the famous “giant *Welwitschia*” individuals in Angola at Flamingo River and Giant Welwitschia South.

**Figure 2:**
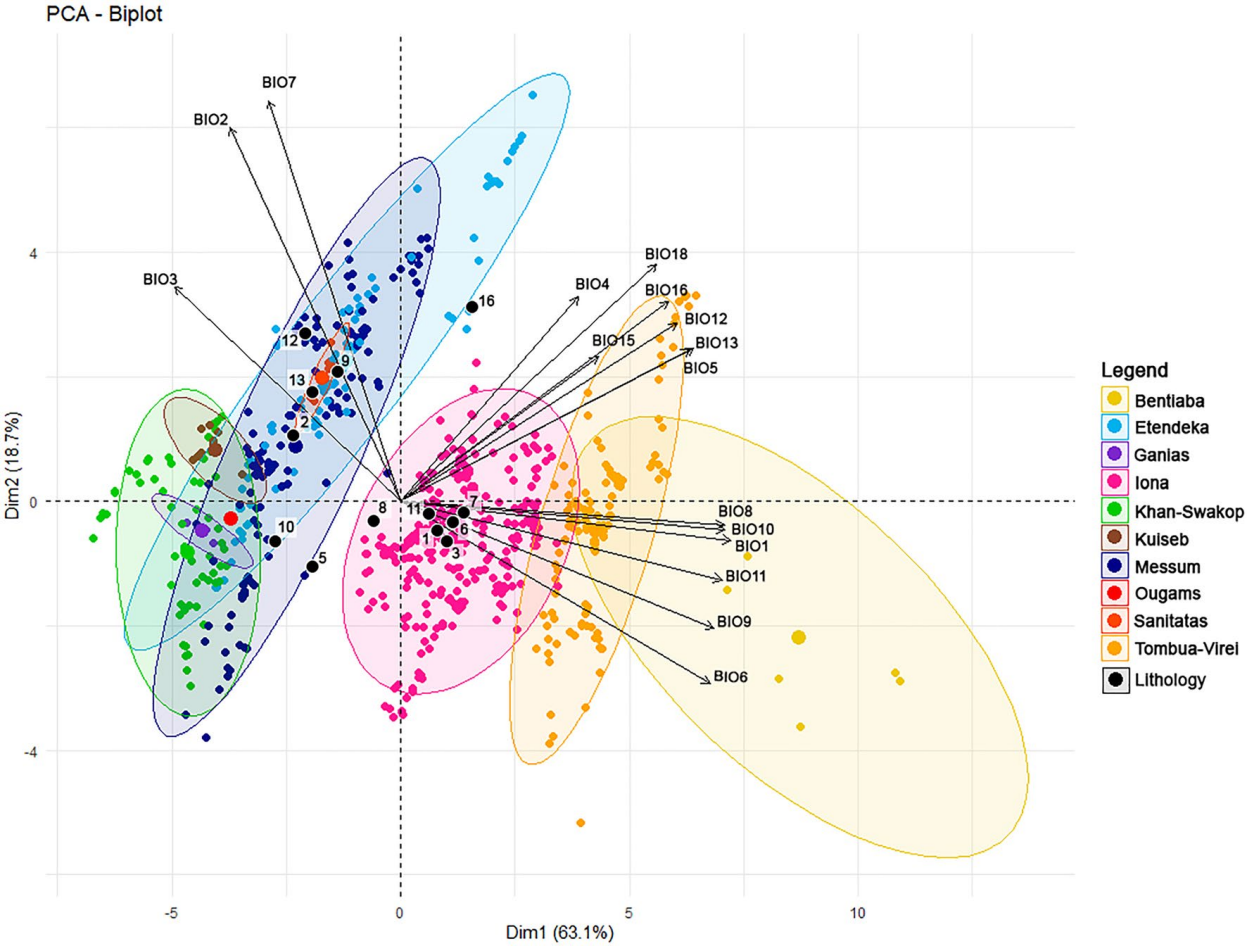
PCA of bioclimate and geology for all observed Welwitschia samples. Colors highlight the different range fragments. Bioclimatic variables: BIO1 = annual mean temperature; BIO2 = mean diurnal range (mean of monthly, max. temp–min. temp); BIO3 = isothermality (BIO2/BIO7) (× 100); BIO4 = temperature seasonality (standard deviation × 100); BIO5 = max. temperature of warmest month; BIO6 = min. temperature of coldest month; BIO7 = annual temperature range (BIO5–BIO6); BIO8 = mean temperature of wettest quarter; BIO9 = mean temperature of driest quarter; BIO10 = mean temperature of warmest quarter; BIO11 = mean temperature of coldest quarter; BIO12 = annual precipitation; BIO13 = precipitation of wettest month; BIO14 = precipitation of driest month; BIO15 = precipitation seasonality (coefficient of variation); BIO16 = precipitation of wettest quarter; BIO17 = precipitation of driest quarter; BIO18 = precipitation of warmest quarter; BIO19 = precipitation of coldest quarter. Lithology: aeolian (1), basalt (2), calcareous (3), complex (5), gneiss (6), granite (7), mica (8), mudstone (9), quartzite (10), calcrete (11), sandstone (12), sand (13), volcanic (16).

We conducted a principal component analysis (PCA) of 16 scaled bioclimatic variables from the WorldClim dataset and a set of 16 lithological units extracted from digitized geological maps of Angola and Namibia. The first two axes of the PCA already explain approximately 81.8% of the variation within the data set (Fig. 2). The observed pattern clearly separates the Angolan and Namibian range fragments on the first two principal axes, while the transitional fragments (Sanitatas, Ougams, Ganias) are separated on the third axis (see Supplementary Figure S1 online). The first principal axis describes a warm–wet (Angola) versus cold–dry (Namibia) gradient, while the second axis reflects the gradient of climatic variability, in particular concerning temperature range. The third axis only represents 8.3% of the total variation, yet the transitional fragments are clustered at the lower end of the axes, where the bioclimatic condition is characterized by low variability in annual and daily temperature (BIO2, 4, and 7). We also conducted a multivariate group test (ANOSIM) to verify statistical differences in bioclimatic composition among the fragments. The R-statistic of 0.55 (*p* < 0.001) confirms the visually observed separation of the fragments with particular overlap between the two groups. Finally, we conducted a Kruskal–Wallis test and Nemenyi post hoc test to analyze the differences among the range fragments in each environmental parameter (Table 3). The overall pattern supports the findings of the PCA.

**Table 3:** Nonparametric Kruskal–Wallis post hoc comparisons between the eight range fragments for bioclimate. The table shows the median (MED) and interquartile range (IQR) for the bioclimate parameters. Small letters indicate similarity between groups identified using a Nemenyi post hoc test. Chi^2^ is the Kruskal–Wallis overall test statistic, p.val is the p-value in asterisks. Note the legend below the table. For bioclimatic parameter abbreviations, see Fig. 2.

	Bentiaba		Tombua–Virei		Iona		Sanitatas		Ougams		Ganias		Etendeka			Messum			Khan–Swakop			Kuiseb				
Parameter	MED	IQR	MED	IQR	MED	IQR	MED	IQR	MED	IQR	MED	IQR	MED		IQR	MED		IQR	MED		IQR	MED		IQR	Chi^2^	p-val
Bio1	^ab^25.04	0.32	^a^24.58	0.19	^b^23.04	0.96	^cd^20.52	0.14	^cd^20.05	0.00	^cd^19.63	0.07	^c^21.15		0.99	^c^21.04		0.97	^d^20.49		0.19	^cd^21.04		0.31	844.07	***
Bio2	^a^11.24	2.55	^a^14.40	0.68	^a^14.43	0.82	^bc^15.40	0.06	^abc^14.91	0.00	^abc^14.69	0.08	^bc^15.98		0.87	^bc^15.96		1.25	^b^15.45		1.19	^c^17.07		0.68	559.79	***
Bio3	^a^64.61	5.08	^a^70.79	1.09	^b^71.58	1.14	^abc^71.37	0.12	^bcdef^73.44	0.00	^bcdef^73.46	0.13	^cd^72.84		1.92	^de^73.26		1.47	^f^73.66		0.60	^ef^73.92		0.40	537.32	***
Bio4	^ab^227.74	0.73	^a^221.30	3.68	^cd^214.82	10.04	^e^198.60	3.07	^cde^201.86	0.00	^ce^200.70	0.57	^bd^214.58		17.34	^cd^212.48		10.86	^e^203.95		7.00	^abcd^216.83		3.06	389.67	***
Bio5	^abc^32.80	0.55	^a^33.60	0.50	^bd^31.80	1.00	^e^30.25	0.20	^defg^29.40	0.00	^ef^28.80	0.00	^cfg^31.10		1.45	^cfg^31.30		1.38	^e^30.50		0.80	^bcdg^31.80		0.30	601.84	***
Bio6	^ab^15.40	1.50	^a^13.25	0.30	^bc^11.80	0.90	^de^8.70	0.10	^cde^9.10	0.00	^de^8.80	0.07	^de^9.50		0.40	^d^9.60		0.30	^e^9.40		0.70	^de^9.10		0.70	858.26	***
Bio7	^a^17.40	2.40	^a^20.30	0.80	^a^20.20	0.90	^bc^21.60	0.10	^abc^20.30	0.00	^ab^20.00	0.07	^c^21.80		1.43	^c^21.80		1.30	^b^21.00		1.40	^c^23.00		0.90	524.59	***
Bio8	^a^27.15	0.38	^a^26.49	0.17	^b^24.97	0.93	^cd^22.33	0.19	^cde^22.27	0.00	^cde^21.85	0.06	^ce^23.38		1.18	^e^23.60		0.96	^d^22.95		0.33	^cde^23.33		0.33	797.41	***
Bio9	^ab^23.70	1.33	^a^23.27	0.26	^b^21.53	1.05	^c^17.60	0.20	^bcd^18.27	0.00	^cd^17.78	0.05	^cd^18.16		0.51	^d^18.67		0.72	^c^17.97		0.23	^cd^18.38		0.37	872.74	***
Bio10	^a^27.15	0.38	^a^26.63	0.22	^b^25.03	0.88	^cd^22.33	0.19	^cde^22.27	0.00	^cde^21.85	0.06	^ce^23.38		1.18	^e^23.60		0.96	^d^22.95		0.33	^cde^23.67		0.23	810.65	***
Bio11	^ab^21.75	0.35	^a^21.35	0.20	^b^19.97	0.97	^cd^17.60	0.20	^cd^17.33	0.00	^cd^16.92	0.07	^cd^18.10		0.70	^c^18.22		0.68	^d^17.97		0.27	^cd^18.38		0.37	844.48	***
Bio12	^ab^153.00	30.50	^abc^109.00	38.50	^ac^102.00	22.00	^b^143.50	22.25	^cde^63.00	0.00	^cde^61.00	2.00	^ac^98.00		50.25	^d^55.00		32.75	^e^33.00		10.75	^de^47.00		5.00	584.77	***
Bio13	^a^61.00	15.00	^abc^39.00	9.75	^d^33.00	7.00	^ab^44.50	5.25	^bcdef^24.00	0.00	^cdef^24.00	0.50	^d^33.00		13.25	^e^21.00		10.00	^f^13.00		2.75	^ef^15.00		2.00	617.96	***
Bio15	^a^135.91	6.23	^a^122.84	1.65	^b^118.78	2.38	^bc^117.80	0.96	^a^126.56	0.00	^a^127.98	1.96	^a^122.63		1.85	^a^123.02		4.67	^c^109.91		3.19	^c^102.71		5.04	673.8	***
Bio16	^ab^111.00	23.50	^ac^80.00	27.75	^ac^76.00	16.00	^b^106.50	14.75	^acde^52.00	0.00	^cde^51.00	1.50	^ac^75.50		36.25	^d^45.00		26.00	^e^27.00		7.75	^de^35.00		4.00	552.01	***
Bio18	^a^111.00	23.50	^ab^75.00	22.00	^c^64.00	13.00	^a^106.50	14.75	^abcde^52.00	0.00	^bcde^51.00	1.50	^ab^75.50		36.25	^d^45.00		26.00	^e^27.00		7.75	^de^30.00		4.00	534.03	***

*** < 0.001, ** < 0.01, * < 0.05, n.s. = not significant.

### Population genetic analyses

We used samples from 17 populations (Fig. 1a, Table 1, in the text referred to by the combination of range fragment and population number, e.g. Etendeka-8.1) representing all range fragments across the whole range in Angola and Namibia to study the genetic background of *Welwitschia* with six SSR markers.

**Figure 3:**
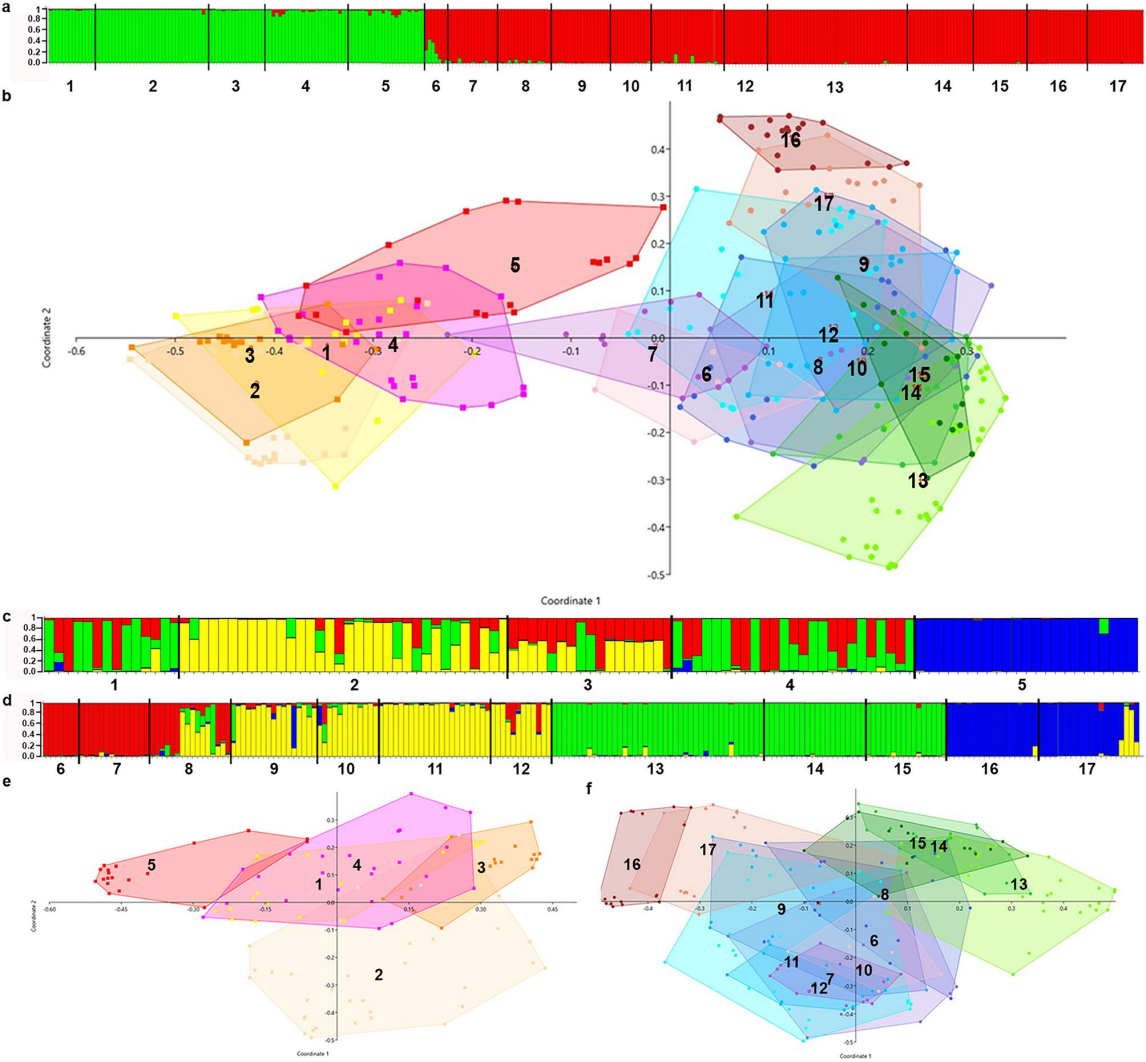
PCoA and gene pools of SSR (population numbers as in Fig. 1a). (**a**) Structure analysis (K = 2) of all studied populations; (**b**) PCoA (Dice) of all 17 populations; (**c**) structure analyses (K = 4) of *W. m. ssp. mirabilis*; (**d**) structure analysis (K = 4) of *W. m. ssp. namibiana*; (**e**): PCoA (Dice) of the five populations of *W. m. ssp. mirabilis*; (**f**): PCoA (Dice) of the 12 populations *W. m. ssp. namibiana*.

At the largest spatial scale (Fig. 3b), PCoAs of all 331 *Welwitschia* individuals analyzed with six SSRs clearly separated a northern subgroup (populations 1–5 in Angola and northern Namibia, on the left in Fig. 3) from a southern subgroup (populations 6–17 in Namibia, on the right in Fig. 3), irrespective of the algorithm applied. The comparison of all populations calculated with the one-way PERMANOVA shows significant differences (F = 11.86, *p* < 0.05) among most of the populations. Only populations 10 and 12 (*p* < 0.33) were not significantly different (Supplementary Tables S1 and S2).

A combined structure and structure harvester analysis of the same sample set also resulted in a “best K” of two, dividing the metapopulation of *Welwitschia* into two clearly separated and almost pure gene pools (Fig. 3a) without stronger gene flow between them. The split between the northern and southern subgroups is located in Namibia at 18.7°S, between the Ougams-5.2 and Ganias-6 populations (the bold white bar in Fig. 1a). Only one population of the southern subgroup (Etendeka-7) shows a weak genetic relationship to the northern subgroup (Fig. 3b).

### Differentiation within the northern subgroup

Looking deeper into the genetic structure of the two subgroups, we divided the sampling set according to the two gene pools, and we reanalyzed the northern and southern populations separately. The structure analysis of 112 plants of the northern subgroup (Fig. 3c) proposed four different gene pools (best K = 4), which partly correspond to geographic patterns (Fig. 1a). Especially, the populations of the Kaoko range fragment (Sanitatas-5.1 and Ougams-5.2) possess a unique gene pool (blue in Fig. 3c) and clusters separately at the left margin in the PCoA (3e). This population is located in northern Namibia, where it is geographically separated by 136 km from the large Iona range fragment in Angola.

Most of the individuals of population 2 are assigned to the same gene pool (Fig. 3c, yellow) and cluster separately in the PCoA (3e), whereas other individuals are assigned to other gene pools (3c, red and green). Population Tombua-Virei-2 is located at the western coastal margin of the large Tombua–Virei range fragment, closest to Welwitsch’s type locality (Fig. 1a). The populations 1, 3, and 4 are even more strongly assigned to combinations of three gene pools. These populations overlap significantly in ordination owing to smaller genetic distances between individuals (Fig. 3e). All populations show significant Bonferroni-corrected p-values (F = 6.83, *p* < 0.001) in a PERMANOVA comparison (Supplementary Tables S1 and S3).

### Differentiation within the southern subgroup

The PCoA patterns of the southern taxon populations are shown in Fig. 3f. The large Etendeka range fragment with populations Etendeka-8 and Etendeka-9 is located in the center. Closely attached are the more coastal populations Etendeka-6 and Etendeka-7), and Messum-10, 11 and 12. In contrast, populations Kuiseb-16 and Kuiseb–17 of the Kuiseb range fragment cluster separately at the upper left corner of the PCoA, while populations Khans-Swakop-13 to Khan-Swakop–15 of the Khan–Swakop range fragment are grouped separately at the upper right corner. As in the northern subgroup, all populations of the southern subgroup differ significantly (F = 13.06, *p* < 0.05) in a pairwise comparison of the PERMANOVA test (Supplementary Tables S1 and S4). The structure analysis of the southern subgroup revealed a best K of four different gene pools. These are presented in Fig. 3d. The populations of the Khan–Swakop range fragment (Khan-Swakop-13 to 15) and Kuiseb range fragment (Kuiseb-16 and Kuiseb–17) in the south are assigned to one unique gene pool (Fig. 3d: Khan–Swakop in green, Kuiseb in blue). The Khan–Swakop and Kuiseb fragments are genetically and geographically well separated from the large central Etendeka–Messum aggregate with gap widths of 83 km and 115 km, respectively. In contrast, the gene pool of Ganias-7 is also found in the western parts of the large Etendeka range fragment.

## Discussion

### Diversity within the refuge

The extant *Welwitschia mirabilis* has developed considerable genetic diversity within the refuge. Our results support the existence of two well-separated taxa within *Welwitschia*, located in both the northern and southern parts of the total range, as proposed by^7^. The gene-flow barrier is located in Namibia at 18.7°S between the Ougams and Ganias range fragments. The structure analysis (Fig. 3a) reveals two separate gene pools with no gene flow between them. This sharp discontinuity is in contrast to their moderate spatial isolation: the nearest range fragments are only 65 km away. At 18.7°S there is no obvious geographical barrier such as a mountain range. Moreover, the first two principal axes of the PCA of 19 scaled bioclimatic variables and geological units indicate that the environmental conditions of the Sanitatas and Ougams range fragments are similar to the other Namibian range fragments (Fig. 2). On the third PCA axis, the Sanitatas range fragment is distinctive for having slightly less variability than most Namibian samples and a slightly cooler and drier climate than the Angolan samples.

However, the location of the discontinuity coincides with an important biogeographical limitation, namely the southern boundary of the Kaokoveld desert ecoregion^26^. It should also be noted that this barrier is equidistant to the range fragments forming the largest aggregations by area in the north (Iona and Tombua–Virei) and south (Etendeka and Messum). It is possible that these major gene pools in the past were the only to survive and later expanded equally into the intermediate space. Further research is needed to explain this biogeographical boundary with regards to *Welwitschia* and other taxa.

Although these data clearly show geographic isolation, at present there is no evidence of reproductive isolation. Therefore, the two taxa are correctly regarded as subspecies of *Welwitschia mirabilis*, with *ssp*. *namibiana* occurring in Namibia south of 18.7° and the typical *ssp*. *mirabilis* found in Angola and the northwestern part of Namibia north of 18.7°.

We found further genetic diversity within each of the two subspecies. The structure analyses identified up to three gene pools for individuals from both subspecies in the large central range fragments (Fig. 3c,d). In contrast to this higher diversity, individuals in the majority of the smaller and more isolated range fragments were assigned to only one gene pool. The observable pattern of partly unique gene pools for subspecies populations in geographically isolated range fragments such as Kaoko, Khan–Swakop, and Kuiseb could indicate a long history of genetic isolation along a north–south corridor. This hypothesis requires further research with more genetic markers.

### The ecological niche of Welwitschia and the disjunct subunits

Compared to the almost global distribution of Welwitschiaceae during the Aptian^3^, the present range of *Welwitschia mirabilis* is limited to the northern part of the Namib Desert. The range is climatically defined by precipitation in the summer months, with a MAP of 20–200 mm. The highest aridity is found in the southernmost parts of the range. The western half of the range receives additional moisture in the form of fog, which increases in amount, frequency, and salinity toward the coast.

The absolute moisture limit is clearly associated with the presence of arid savanna vegetation. *Welwitschia* is unable to compete with woody arid savanna plants, especially *Acacia reficiens*, *Acacia mellifera*, *Acacia tortilis*, *Colophospermum mopane*, and *Salvadora persica*. Mixed stands of these woody plants are only found in arid river environments, which offer a local mixture of more or less arid sites depending on the history of floods, sedimentation, and terrace erosion. Rarely, a smaller-scale mosaic of moister and drier habitats allows an interdigitation of *Welwitschia mirabilis* stands, woody acacias, and *Colophospermum* trees at the 10–100 m scale, as for example in the western part of Virei and Khorixas. In contrast, *Welwitschia* is able to share habitats with woody species of *Commiphora*, *Calicorema capitata*, *Zygophyllum stapffii*, *Arthraerua leubnitziae*, and perennial grasses.

The aridity limit is less clearly defined and will depend on coastal factors such as fog, salinity, wind speed, sand blasting, and cooler temperatures. In several range fragments (Tombua, Ougams, Uniab, Messum, Khan–Swakop) the aridity limit toward the coast is defined by the extent of inland gravel sediments in the coastal area.

Fields of sand dunes south of the Kuiseb River and near the coast between Torra Bay and Tombua also form an important limitation.

However, the general climatic niche of 20–200 mm MAP does not explain the disjunct range of *Welwitschia* encompassing a broad, uninterrupted band from the Kuiseb to central Angola.

The PCA of 19 scaled bioclimatic variables and geological units clearly separates the Angolan and Namibian range fragments. But in addition, the different Angolan range fragments are well separated from each other, while in Namibia the Khan–Swakop and Kuiseb subunits differ to some degree from the rest of the Namibian subunits. However, the rest of the Namibian range fragments are quite similar in their bioclimatic parameters.

In some range fragments, the lithology defines sharp boundaries. For example, the sharp boundary at the western edge of the Etendeka range fragment is clearly defined by the western boundary of Etendeka basalt. In fact, all the northern range fragments of *W. m. ssp. namibiana* share a strong though not obligate affinity to Etendeka basalts (Ganias, Etendeka, Messum). In contrast, other range fragments of *Welwitschia* are located on markedly different rock types, including granite, gneiss, mica schist, limestone, and sandstone. In some cases (e.g., Iona) the presence of *Welwitschia* fades out within a geological unit.

The habitat preference of alluvial soils in or adjacent to small dry riverbeds and/or terraces bordering larger dry riverbeds seems to be a unifying factor for many, if not all, *Welwitschia* range fragments. Such small drainage channels attract runoff water and increased infiltration of water during rare rainfall events and allow the soil to retain water within and under its calcrete (and sometimes gypcrete) horizons^13^ during periods without rainfall. After rainfall, small drainage channels especially seem to provide the level of soil moisture preferred by *Welwitschia mirabilis* in the vadose zone. Some of the smaller range fragments and their subunits (Sanitatas, Ougams, Ganias, the Khan gorges, and the plains south of Swakop) could reasonably be understood as interconnected catchment systems. Similarly, the western boundary toward the coast of many range fragments (Tombua, Ougams, Uniab, Messum, Khan–Swakop) is defined by the extent to which inland gravel sediments in catchments or sheetwash plains spread over the coastal area. In some places, especially in the north (e.g., in large parts of Iona and Tombua–Virei), rocky plains with shallow leptosols, often combined with calcrete, are the dominant habitat.

### Adaptive diversification within the refuge?

The data and observations presented here allow the following hypothesis: the observed diversity of *Welwitschia* within the refuge could have evolved there through adaptation to different landscapes. An alternative hypothesis is that the present genetic diversity already existed when *Welwitschia* disappeared elsewhere and became limited to the northern Namib Desert.

The present diversity is minimal. Though the diversification of gene pools is clearly visible at the molecular level, it is hard to distinguish even the two subspecies morphologically in the field, whereas cultivation under identical conditions did allow us to distinguish the two subspecies^7,8^. This relatively low level of diversification could support the hypothesis that only one common ancestor settled the refuge and has since diversified at a slow rate. We did not sample morphological data during our one-off field visits because the extremely variable environmental conditions (e.g., the recent history of drought, herbivore browsing) strongly determined the conditions of the relevant morphological features.

*Welwitschia* plants cannot compete with more modern woody arid-adapted savanna plants, in slightly moister habitats. However, throughout the range, *Welwitschia* plants are able to inhabit the above described niche near dry riverbeds and, especially, small drainage channels that form part of larger catchment systems. Perhaps their potentially very long lifespan is essential and enables *Welwitschia* plants to outcompete other species that establish themselves only during cycles of moist years.

*Welwitschia* populations within the different arid catchments could have evolved adaptations to locally abundant rock types or other habitat conditions, including fog and water storage in gypcretes, for example. After the development of a major gene-flow barrier between the two subspecies, both were able to maintain large core areas, while satellite populations in suitable catchments north and south of the core area could survive and develop individual gene pools. In conclusion, our data and observations allow us to interpret *Welwitschia mirabilis* as a paleoendemic species surviving in an arid refuge where it further adapted to environmental conditions in disjunct range fragments.

### Future research

The preceding hypothesis, derived from investigating genetic divergence according to six SSR markers from all range fragments and niche divergence according to numerous environmental variables, should be tested by other research methods. Analyzing additional chloroplast or nuclear markers would be necessary to confirm our SSR results and should receive highest priority. Similarly, a somewhat higher spatial resolution would be required to better understand the genetic diversity within the largest range fragments. The integration of bioclimatic and geological variables presented here should be expanded to include potentially important variables related to fog, soil properties, small-scale habitat features such as gradients next to small riverbeds, and other species within the relevant plant communities. Utilizing these gradients to better quantify the ecological niche of the subspecies would be a necessity to further support the subspecies hypothesis.

Perhaps the future discovery of fossil sites containing relatives of *Welwitschia* younger than 112 My will give us a more holistic understanding of how this important “living fossil” evolved.

## Material and methods

### Biogeography

In the first author’s project, “Vegetation of the Namib Desert,” a systematic assessment from near Benguela (-12.5°S) to the southern margin of the Knersvlakte (-32°S) allowed us to distinguish among locations where *Welwitschia* was or was not recorded. The field work was accompanied by targeted efforts to find potential *Welwitschia* populations using all available historical and recent information from collections, museums, literature^9,11,12,14,27^, and local people. Records with only broad geographical information were scrutinized during additional field visits. In addition, high-resolution aerial photographs were used to assess the presence of *Welwitschia* in inaccessible regions, especially in Angola^28^, and our own UAV pictures allowed inspection of inaccessible places. At each site, we also recorded all accompanying vegetation within 1000 m^2^, took soil samples, and noted additional environmental parameters.

To better understand the ecology of the fragments, we analyzed their position along the large-scale climate gradient between arid Namibia and southwestern Angola, and we considered the importance of lithology, which might have large-scale consequences for the occurrence pattern. We compiled lithological units from the most recent digital geological maps of Angola and Namibia^29,30^ provided by Geological Services. We assembled the bioclimatic data from the WorldClim V2.1^24^ data set with a resolution of 1 km^2^ per grid cell. These data describe 19 different bioclimatic variables, such as mean annual temperature (BIO1), temperature range (BIO7), and mean annual precipitation (BIO12). For a description of the variables, please refer to https://www.worldclim.org/data/bioclim.html. We extracted bioclimate data for all observations of *Welwitschia* in our database. The parameters BIO14, BIO17, and BIO19 indicate precipitation in the driest or coldest month or quarter. These were constantly zero or had zero variation and were removed in further analysis. For each parameter, we checked for differences between the medians of the ranked bioclimate values for the range fragments using a Kruskal–Wallis test. This nonparametric procedure was necessary because there were strong differences in the number of samples per fragment. We added a Nemenyi post hoc test to identify significant differences among the single-range fragments. To better understand the relationships among the range fragments and with regards to climate and lithology, we additionally conducted a PCA of the dataset. PCA is an eigenvalue-based data-dimension reduction technique that finds major orthogonal gradients in the data and reduces this pattern to so-called principal components or principal axes. The result is then visualized with a bi-plot in which the variables and samples are interpreted relative to their positions on the first two or three principal axes. The data were standardized and analyzed with a correlation instead of a variance–covariance matrix. Finally, we ran a nonparametric multivariate group test to check for the strength of separation between the clusters. We used the ANOSIM procedure, which analyzes differences among the ranks of group centroids for a multivariate data set and effectively measures the overlap among the different clusters. The analysis generates R-statistics and a p-value based on 999 permutations. Values below 0.5 indicate strong overlap among clusters, values below 0.75 indicate some overlap, and values above 0.75 indicate little to no overlap among the different multivariate groups. All analyses were run in R^31^, with the packages asbio^32^, PMCMR^33^, FactoMineR^34^, and vegan^35^.

### Leaf tissue sampling of Welwitschia populations

Small tissue samples of 2 cm^2^ were taken from still-green leaf areas at the outer margin of the living leaf to avoid unnecessary damage. Samples were dried with silica gel in plastic bags. Tissues were taken from 331 individuals, which formed part of 31 sampling areas in 17 populations (Table 1) ranging from seven to 42 individuals per population. At each sampling area, seven to 15 plants were sampled.

### Establishment of SSR markers for Welwitschia mirabilis

All 6.214 W*. mirabilis* ESTs of the Floral Genome Project^36^ were screened for SSR motives with Phobos V3.3.11^37^. The dinucleotide repeats (AG)n, (AT)n, (AC)n, and (CG)n were chosen as SSR motives, and the minimum of n repeats was set to four. Out of 157 identified SSR motives, 16 ESTs were chosen to create SSR markers. Using Primer3 Version 1.1.4.^38^, primers were designed in front of and behind the microsatellite motif to create PCR products between 100 and 350 bp. Six out of 16 primer pairs produced distinct, reproducible, and polymorphic PCR products (Table 4). The SSR loci were also sequenced to furnish proof of the evidence of the microsatellite locus. DNA isolation and quality tests were performed according to Dumolin et al.^39^ with modifications described in^40^. The DNA concentration was measured with a NanoPhotometer (Implen GmbH, Munich, Germany) according to the manufacturer’s instruction and finally adjusted to 20 ng/µL.

### PCR amplification and fragment analyses

The microsatellite loci were amplified as described in^40^ with modifications to the 3500 Genetic Analyzer (Thermo Fisher Scientific, Waltham, MA, USA). All forward primers were labeled either with 6-FAM or HEX (Table 4). The 10-µl PCR mix included 1 × PCR buffer B, 0.2 mM dNTPs, 0.25 U "my-Budget Taq-DNA Polymerase" (from Bio-Budget Technologies GmbH, Krefeld, Germany), 2 µM of each primer, 10% DMSO, and 20 ng DNA. The touchdown PCR was carried out with the following conditions: 95 °C for 3 min, 94 °C for 60 s, 65 °C for 30 s, and 72 °C for 45 s. Annealing temperature was decreased every second cycle for 1 °C to 52 °C. Finally, the cycle number was increased to 16, and the reaction ended with an elongation step at 72 °C for 8 min. The SSR loci were prepared to the manufacturer’s protocol for the 3500 Genetic Analyzer and measured with the GeneScan LIZ 500 dye Size Standard (Thermo Fisher Scientific, Waltham, MA, USA).

**Table 4:** SSR Primers of *Welwitschia mirabilis* with the DNA sequence.

Name	Sequence
Wemi 1 F 6Fam Wemi 1 R	5´-AGC ACA GAA ATC TCC AAA GG 5´-AGC CAG TTA GAG GAG TGA GC
Wemi 8 F 6Fam Wemi 8 R	5´-ACA AAC ACC CAC CTC GTT AT 5´-TCT TCT GTT TTT GGG GTT TC
Wemi 9 F 6Fam Wemi 9 R	5´-AGA CAA GCA CAA ATG CAA GT 5´-AAA AAT TCT TGT AAG TCA TAT GTA GAG
Wemi 11 F Hex Wemi 11 R	5´-TCT ATG CTC CAT TGG GTT TT 5´-AAA TGC AGG GCA TTA AAC AT
Wemi 14 F Hex Wemi 14 R	5′-CTTGAGAGCTAAATGCC 5′-TGAAGAGCCAGTTAGAGGA
Wemi 16 FHex Wemi 16R	5′-CATCAACTCTCTTTGCC 5′-TTTGCGATTGTTGAGGTACT

### Data analyses

All samples were analyzed at least twice, and only reproducible PCR products were included in dominant and codominant analyses. The PCR products were visualized in GeneMapper V 4.1 (Thermo Fisher Scientific, Waltham, MA, USA). For dominant marker analyses, PCR products of the same SSR locus with the same sizes were considered as homologue alleles and coded as “1”; and no PCR products of the same size were coded as “0” in a 1/0 matrix. Questionable sites were treated as missing data and marked with a question mark. Peak shifts in sizes of less than 1 bp were adjusted to the respective allele size manually. For further analyses, all six SSR markers were combined in one 1/0 matrix. Based on first results, the data matrix with all 17 populations was split into two partial matrices: 1) all northern populations of Angola plus the northernmost Namibian populations (populations 1–5), and 2) the remaining Namibian populations (populations 6–17).

PCoAs were run with the combined and two partial 1/0 matrices in PAST V 3.23^41,42^. The algorithm for this metric multidimensional scaling was based on Davis^43^. The PCoAs were estimated using the Sørensen–Dice^44^ coefficient and a transformation exponent of c = 2. Missing data were deleted pairwise and omitted from calculation^41^. The two dimensions with the highest value in an n-dimensional room were chosen to explain the distribution of the genetic characters in a two-dimensional scatterplot. Significant differences among populations of *Welwitschia* were identified by a one-way PERMANOVA^45^ with 9,999 permutations in PAST^41,42^. The Bonferroni-corrected p-values are shown in Supplementary Tables 1–4.

To identify the number of genetic populations (= best K) and the degree of admixture between and within each genetic population of *Welwitschia*, a codominant data matrix was created with allele sizes of each SSR locus coded in base pairs (bp). The complete data matrix was divided into two data matrices as described earlier. For all three SSR data matrices, Bayesian structure analyses were estimated with the program structure 2.3.4^46–48^. To ensure an unbiased best-K estimate of the *Welwitschia* metapopulation, we analyzed all evolution model algorithms offered by the program structure with 30,000 MCMC replicates and a burn-in period of 10,000, a number of putative populations K between 1 and 18, and 30 replications per run. The output of all analyses was visualized by the online program Structure Harvester^49^, which estimates the changing rate in log probabilities between consecutive values of K according to the ad hoc statistic ∆K method^50^. A structure analysis was repeated with the best K, estimated by Structure Harvester with 100,000 MCMC replicates, a burn-in period of 30,000, and 50 replications.

## Supplementary information

Supplementary information.

## Data Availability

Data are available on request from the corresponding author.
