# Supplementary material for: Welwitschia: Phylogeography of a living fossil, diversified within a desert refuge
Source: Sci Rep. 2021 Jan 27;11:2385. doi: 10.1038/s41598-021-81150-6 (PMC7840819; doi:10.1038/s41598-021-81150-6)
Supplement: Supplementary file 1 — Supplementary information. [file 41598_2021_81150_MOESM1_ESM.pdf]

Supplementary Information for "*Welwitschia*: Phylogeography of a living fossil, diversified within a desert refuge"

Authors

Norbert Jürgens *Correspondence to norbert.juergens@t-online.de, Imke Oncken, Jens Oldeland, Felicitas Gunter, Barbara Rudolph

Institute of Plant Sciences and Microbiology, Team Biodiversity, Evolution and Ecology of Plants (BEE), University of Hamburg, Ohnhorststrasse 18, 22609 Hamburg, Germany+

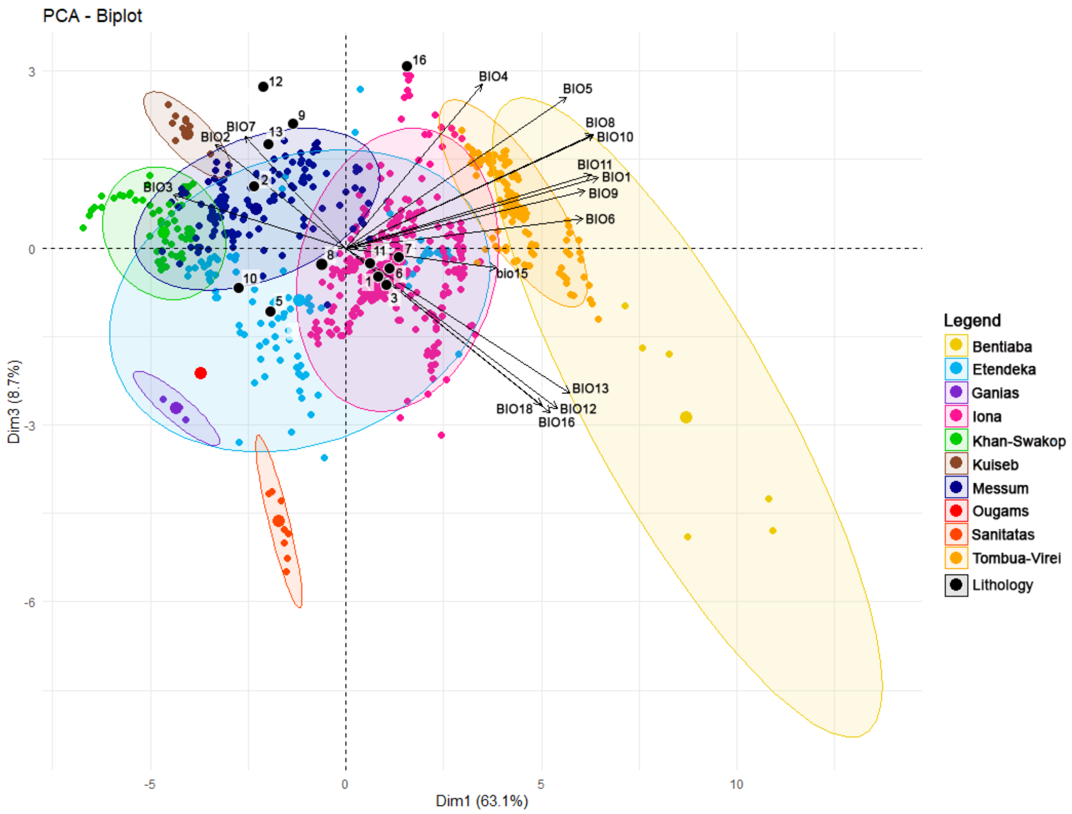

Supplementary Figure S1 PCA of bioclimate and geology for all observed *Welwitschia* samples along first and third axes. Colors highlight the different range fragments. Bioclimatic variables: BIO1 = Annual Mean Temperature, BIO2 = Mean Diurnal Range (Mean of monthly (max temp - min temp)), BIO3 = Isothermality (BIO2/BIO7) (×100), BIO4 = Temperature Seasonality (standard deviation ×100), BIO5 = Max Temperature of Warmest Month, BIO6 = Min Temperature of Coldest Month, BIO7 = Temperature Annual Range (BIO5-BIO6), BIO8 = Mean Temperature of Wettest Quarter, BIO9 = Mean Temperature of Driest Quarter, BIO10 = Mean Temperature of Warmest Quarter, BIO11 = Mean Temperature of Coldest Quarter, BIO12 = Annual Precipitation, BIO13 = Precipitation of Wettest Month, BIO14 = Precipitation of Driest Month, BIO15 = Precipitation Seasonality (Coefficient of Variation), BIO16 = Precipitation of Wettest Quarter, BIO17 = Precipitation of Driest Quarter, BIO18 = Precipitation of Warmest Quarter, BIO19 = Precipitation of Coldest Quarter. Lithology: 1 Aeolian, 2 Basalt, 3 Calcareous, 5 Complex, 6 Gneiss, 7 Granite, 8 Mica, 9 Mudstone, 10 Quartzite, 11 Calcrete, 12 Sandstone, 13 Sand, 16 Volcanic.

Supplementary Table S1 Statistics of the one-way PERMANOVA between all populations (17), populations of the northern subspecies (1 to 5) and the southern subspecies (6 to 17) calculated with 9999 permutations

	Sum of squares	Mean sum of squares within-group	F	$P_{(same)}$
All 17 populations	2310	1462	11.86	0.0001
W. m. ssp. mirabilis populations (1-5)	917.9	735.1	6.83	0.0001
W. m. ssp. namibiana populations (6-17)	1235	755.4	13.06	0.0001

Supplementary Table S2 Bonferroni corrected p-values of the one-way PERMANOVA between individuals of all population from Angola (1 to 5) and Namibia (6 to 17).

Populations	1	2	3	4	5	6	7	8	9	10	11	12	13	14	15	16
2	0.0136															
3	0.0136	0.0136														
4	0.0136	0.0136	0.0136													
5	0.0136	0.0136	0.0136	0.0136												
6	0.0136	0.0136	0.0272	0.0136	0.0136											
7	0.0136	0.0136	0.0136	0.0136	0.0136	0.0272										
8	0.0136	0.0136	0.0136	0.0136	0.0136	0.0136	0.0136									
9	0.0136	0.0136	0.0136	0.0136	0.0136	0.0136	0.0136	0.0272								
10	0.0136	0.0136	0.0136	0.0136	0.0136	0.0136	0.0136	0.0136	0.0136							
11	0.0136	0.0136	0.0136	0.0136	0.0136	0.0136	0.0136	0.0136	0.0136	0.0136						
12	0.0136	0.0136	0.0136	0.0136	0.0136	0.0272	0.0136	0.0136	0.0136	**0.3264**	0.0136					
13	0.0136	0.0136	0.0136	0.0136	0.0136	0.0136	0.0136	0.0136	0.0136	0.0136	0.0136	0.0136				
14	0.0136	0.0136	0.0136	0.0136	0.0136	0.0136	0.0136	0.0136	0.0136	0.0136	0.0136	0.0136	0.0136			
15	0.0136	0.0136	0.0136	0.0136	0.0136	0.0272	0.0136	0.0136	0.0136	0.0136	0.0136	0.0136	0.0136	0.0136		
16	0.0136	0.0136	0.0136	0.0136	0.0136	0.0136	0.0136	0.0136	0.0136	0.0136	0.0136	0.0136	0.0136	0.0136	0.0136	
17	0.0136	0.0136	0.0136	0.0136	0.0136	0.0136	0.0136	0.0136	0.0136	0.0136	0.0136	0.0136	0.0136	0.0136	0.0136	0.0136

Supplementary Table S3 Results of the one-way PERMANOVA between populations 1 to 5 of the northern subspecies *W. m. ssp. mirabilis*. Shown are the Bonferroni-corrected p-values.

Populations	1	2	3	4
2	0.001			
3	0.001	0.001		
4	0.001	0.001	0.001	
5	0.001	0.001	0.001	0.001

Supplementary Table S4 Bonferroni corrected p-values after the pairwise comparison of the one-way PERMANOVA between populations 6 to 17 of the southern subspecies *W. m. ssp. namibiana*.

Population	6	7	8	9	10	11	12	13	14	15	16
7	0.0066										
8	0.0066	0.0066									
9	0.0066	0.0066	0.0132								
10	0.0066	0.0066	0.0198	0.0066							
11	0.0066	0.0066	0.0066	0.0066	0.0066						
12	0.0066	0.0066	0.0066	0.0066	0.0396	0.0066					
13	0.0066	0.0066	0.0066	0.0066	0.0066	0.0066	0.0066				
14	0.0066	0.0066	0.0066	0.0066	0.0066	0.0066	0.0066	0.0066			
15	0.0066	0.0066	0.0066	0.0066	0.0066	0.0066	0.0066	0.0066	0.0066		
16	0.0066	0.0066	0.0066	0.0066	0.0066	0.0066	0.0066	0.0066	0.0066	0.0066	
17	0.0066	0.0066	0.0066	0.0066	0.0066	0.0066	0.0066	0.0066	0.0066	0.0066	0.0066
